# A study protocol for a randomized controlled trial of an anti-inflammatory nutritional intervention in patients with fibromyalgia

**DOI:** 10.1186/s13063-021-05146-3

**Published:** 2021-03-09

**Authors:** Ana Rita Silva, Alexandra Bernardo, Maria Fernanda de Mesquita, José Vaz Patto, Pedro Moreira, Maria Leonor Silva, Patrícia Padrão

**Affiliations:** 1 Faculdade de Ciências da Nutrição e Alimentação, Universidade do Porto, Rua Dr. Roberto Frias, 4200-465 Porto, Portugal; 2 Centro de Investigação Interdisciplinar Egas Moniz (CiiEM), Instituto Universitário Egas Moniz, Almada, Portugal; 3 Instituto Português de Reumatologia (IPR), Rua Beneficiência 7, 1050-042 Lisbon, Portugal; 4 EPIUnit, Instituto de Saúde Pública, Universidade do Porto, Rua das Taipas, n° 135, 4050-600 Porto, Portugal; 5 Centro de Investigação em Atividade Física, Saúde e Lazer, Universidade do Porto, R. Dr. Plácido da Costa 91, 4200-450 Porto, Portugal

**Keywords:** Fibromyalgia, Diet, Anti-inflammatory, FODMAPs, Pain, Quality of life, Randomized controlled trial

## Abstract

**Background:**

This study aims to analyze the effects of a potentially anti-inflammatory nutritional intervention in disease assessment parameters, inflammatory markers, and quality of life of fibromyalgia (FM) patients.

**Methods:**

A sample of 100 female patients diagnosed with FM, followed up at Portuguese Institute of Rheumatology (IPR) in Lisbon, is being randomly allocated in two groups. Patients in the intervention group are adopting an anti-inflammatory diet, characterized by the exemption of the intake of foods containing gluten, dairy, sugar, and ultra-processed foods, during 3 months. During the first month, a low fermentable oligo-, di-, and monosaccharides and polyols (FODMAPs) diet is implemented, along with the anti-inflammatory diet, followed by the reintroduction of all fruits and vegetables over a consecutive period of 2 months. Patients in the control group are adopting a diet based on general recommendations for healthy eating. The outcomes are pain, fatigue, quality of sleep, quality of life, gastrointestinal symptoms, and inflammation. Before and after the 3 months intervention, and also 1 month after beginning the intervention, the following questionnaires are applied: Revised Fibromyalgia Impact Questionnaire, visual analog pain scale, Brief Pain Inventory,visual analog scale from a list of common gastrointestinal and extraintestinal symptoms in FM, Short Form 36, Fatigue Severity Survey, and Pittsburg Sleep Quality Index. Ultra-sensitive serum C-reactive protein, eritrocyte sedimentation rate, and interleukin-8 are determined. Age, physical activity, anthropometric parameters, and body composition are being collected. Student’s *t* test will assess the association between the disease evaluation parameters, the inflammatory markers, and the dietary interventions.

**Discussion:**

The results of this study are expected to determine whether a change in patient nutrition helps to alleviate symptoms, which would optimize medical intervention.

**Trial registration:**

www.ClinicalTrials.gov NCT04007705. Registered on July 5, 2019.

**Supplementary Information:**

The online version contains supplementary material available at 10.1186/s13063-021-05146-3.

## Background

Fibromyalgia (FM) is a chronic non-degenerative disease of unknown etiology, with a prevalence range between 0.5 and 2% worldwide [1], 2.1% (95% CI 2.0–2.2) in men and 3.6% (CI 95% 3.5–3.7) in women [2]. In Portugal, the estimated prevalence is 1.7% [3]. The main symptoms of the disease are musculoskeletal pain and chronic fatigue, in addition to nonrestorative sleep, morning stiffness, depression, anxiety [1], and gastrointestinal (GI) symptoms similar to irritable bowel syndrome (IBS) [4]. Medical therapy consists mainly in analgesic, muscle relaxants, and non-steroids anti-inflammatory drugs (NSAID), but it seems not to completely resolve the symptoms of the disease [1, 5].

Recently, several authors showed an association between FM and dysbiosis [6, 7], and in particular with small intestinal bacterial overgrowth (SIBO) [8, 9], characterized by the inappropriate colonization of the distal small bowel with colonic bacteria [10]. A clinical trial with 38 FM women showed that a low ingestion in fermentable oligo, di-, and monosaccharides and polyols (FODMAPs) could improve SIBO, decreasing pain associated with FM, fatigue, gastric pain, and intestinal changes after 4 weeks [11].

Furthermore, other studies revealed a presence of intestinal inflammation [4, 12–14], through a plasma pro-inflammatory cytokines increase [15–17], particularly interleukin (IL)-6 and IL-8 [16, 17], suggesting a low grade inflammation in these patients, associated with dysbiosis [6, 7]. Literature suggests that foods with inflammatory potential, as the ones described in “Dietary Inflammatory Index” [18, 19] could have a critical role in FM symptoms. Additionally, it is also known the pro-inflammatory effect of gluten [20], dairy [21], and ultra-processed foods [22], and on the other hand, the anti-inflammatory potential of omega 3 [23] and antioxidants [24].

In fact, in a systematic review conducted by our team, it was reported that pain and functional repercussion in FM along with quality of life [25–27], quality of sleep [26], anxiety [27], depression [27, 28], and inflammatory biomarkers [28] seem to improve with a hypocaloric diet [27, 28], a raw vegetarian diet [25, 26] or a low FODMAPs diet [11]. However, the existing clinical trials on this subject are scarce and low quality, which does not allow conclusions to be drawn [29]. Additionally, to our knowledge, a nutritional approach involving a combination of several anti-inflammatory dietary factors has never been designed.

Taking those findings together, it seems relevant to test the hypothesis that a dietary intervention which includes potentially anti-inflammatory foods and excludes the potentially pro-inflammatory ones, and that simultaneously allows an optimization of the intestinal microbiota, could reduce intestinal inflammation and dysbiosis, and consequently improve the FM patient’s reported outcomes (PRO).

## Methods and analysis

The study protocol was developed considering the SPIRIT checklist (Additional file 1) and guidelines and is registered in www.ClinicalTrials.gov (NCT04007705).

The study aims to analyze the effects of a potentially anti-inflammatory and low FODMAPs diet, compared to healthy eating recommendations, in disease assessment parameters, namely pain, fatigue, sleep quality, and GI alterations, in inflammatory markers and quality of life in FM patients.

### Study design

A randomized controlled clinical trial, blind to patients, has started in April 2019 at the Portuguese Institute of Rheumatology [Instituto Português de Rematologia (IPR)] in Lisbon. All women diagnosed with FM followed-up at the IPR, with a medical appointment scheduled between February 2019 and December 2020, are being invited to participate in the study. The recruitment is being performed as the patients are identified in the appointment.

### Study setting

After eligibility criteria confirmation and informed consent applied (Additional file 2), participants are being allocated to intervention or control group. Allocation of participants is performed using systematic procedures. Participants were sequentially assigned to intervention or control group as they were recruited. Due to the nature of the intervention, the allocation of experimental groups is blind to patients but not to researchers, as they will then apply the appropriate dietary plan. Each participant is given a code, to ensure anonymity and confidentiality of collected data.

### Sample size

In order to define the sample size required for the study and to give a statistical power of 80%, G-Power Software version 3.1.9.4 revealed that, for a desirable effect size of 50%, a minimum sample size of 45 individuals is required. In order to prevent follow-up losses, the target sample size is *n* = 100.

### Participant characteristics and eligibility criteria

Inclusion criteria are:

1. Female adults, aged over 18 and under 75 years old;
2. Diagnosis of FM performed by the physician, according to the Rome III criteria of the American College of Rheumatology, revised in 2010;
3. Ability to read and sign the informed consent; and
4. Stable dose therapy within 4 weeks before the study begins.

Exclusion criteria are:

1. Patients with pathologies that prevent to follow the dietary intervention;
2. Patients currently undergoing lactation or pregnancy;
3. Prior or current clinical history of abuse of drug or other substances;
4. Change of therapy during the intervention period;
5. Presence of other inflammatory diseases; and
6. Uncontrolled medical conditions (e.g., diabetes mellitus, heart disease, renal failure, neoplastic diseases, liver diseases).

### Intervention

Patients are being contacted to schedule the first phase of the study (M0). During the first meeting with the researchers, a blood sample is collected, and the evaluation questionnaires are fulfilled. Additionally, anthropometric parameters (weight, height, and waist perimeter) and body composition are assessed, using a bio-impedance scale. The diet meal plan is determined by an investigator team nutritionist, according to the allocated group, taking into account basal metabolic rate, physical activity, lifestyle, food habits, and preferences of the patient, in order to ensure its feasibility. After 3 months of intervention, patients from both groups meet the researcher in order to perform a new blood collection, to assess weight and body composition and to complete all the evaluation questionnaires.

### Dietary interventions specifications

The intervention group (G1) is adopting an anti-inflammatory diet, which is characterized by the exclusion of potentially inflammatory foods, namely gluten, dairy, sugar, and ultra-processed foods, over a consecutive period of 3 months. During the first month, a low FODMAPs diet is being implemented along with the anti-inflammatory diet, followed by the reintroduction of all fruits and vegetables over a consecutive period of 2 months, for a total of 3 months of intervention. The control group (G2) is adopting a diet based on recommendations for healthy eating in accordance with the World Health Organization (WHO) [30]. Both diets are determined by a nutritionist investigator during nutrition consultations using a leaflet, to help compliance. Examples of recipes are being delivered to help patients to comply with the outlined dietary plan. A table of foods to consume and to avoid is being provided to participants belonging to the intervention group during low FODMAPs diet phase (Additional file 3).

### Adherence

During the intervention period, patients are being monitored every 15 days, by telephone, in order to assess compliance and any change regarding the inclusion and exclusion criteria, as well as to clarify any question about the intervention. Biweekly phone contacts are made in order to monitor the compliance with the recommendations. Participants are asked to fulfill a food diary of the 3 previous days to phone contact, and energy, macro, and micronutrients intake are then calculated for both groups. In addition, time of different meals is also analyzed. The Food Processor Software (version 11.2.274) is being used to analyze food records.

**Fig. 1:**
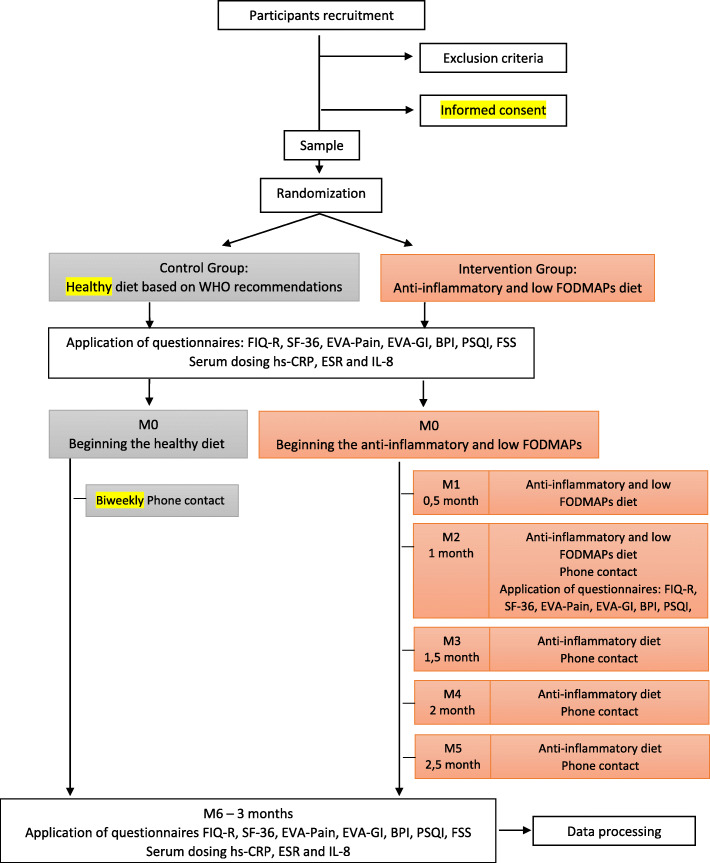
Experimental design of the randomized controlled clinical trial. Note: FODMAPs, fermentable oligo-, di-, and monosaccharides and polyols; FIQ-R, Revised Fibromyalgia Impact Questionnaire; SF-36, Short Form 36; EVA-Pain, visual analog pain scale; EVA-GI, visual analog scale from a list of common gastrointestinal and extraintestinal symptoms in FM; BPI, brief pain inventory; PSQI, Pittsburg Sleep Quality Index; FSS, Fatigue Severity Survey; hs-CRP, serum C-reactive protein; ESR, eritrocyte sedimentation rate; IL-8, interleukin-8

**Fig. 2:**
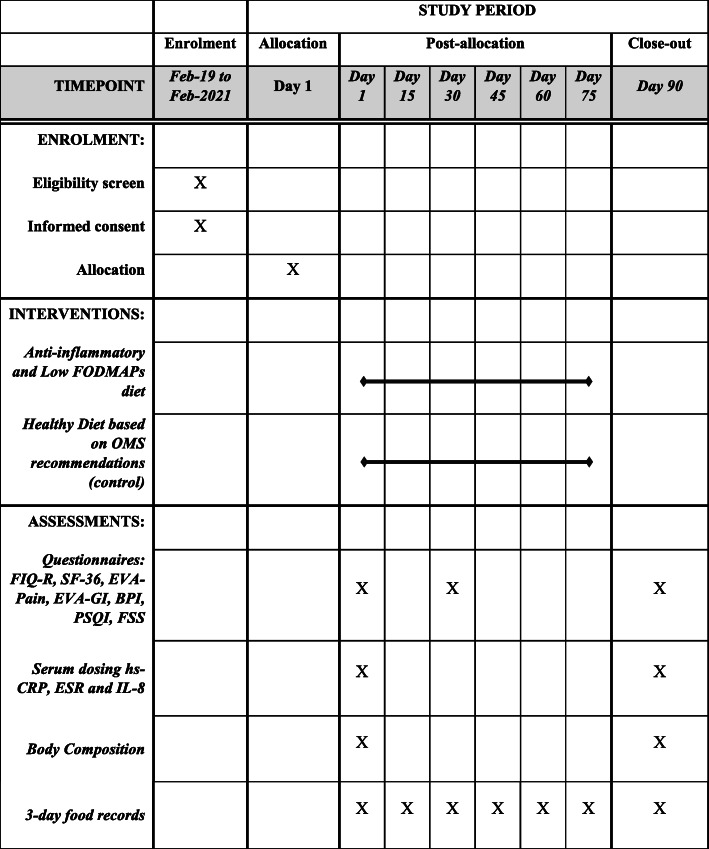
Schedule of enrolment, interventions, and assessments, according to SPIRIT guidelines. Note: FODMAPs, fermentable oligo-, di-, and monosaccharides and polyols; FIQ-R, Revised Fibromyalgia Impact Questionnaire; SF-36, Short Form 36; EVA-Pain, visual analog pain scale; EVA-GI, visual analogue scale from a list of common gastrointestinal and extraintestinal symptoms in FM; BPI, brief pain inventory; PSQI, Pittsburg Sleep Quality Index; FSS, Fatigue Severity Survey; hs-CRP, serum C-reactive protein; ESR, eritrocyte sedimentation rate; IL-8, interleukin-8

The experimental design of the present study is shown schematically in Figs. 1 and 2.

### Intervention group

#### Anti-inflammatory diet

The anti-inflammatory diet is characterized by the exclusion of potential inflammatory foods, such as gluten, dairy, free sugar, and ultra-processed food, rich in sugar, hydrogenated fat, and food additives. Despite the controversy surrounding the ingestion of these foods, some authors defend the existence of an association of these with an increase in serum C-reactive protein (CRP) [18, 31, 32] and various inflammatory diseases [33], including rheumatic diseases [34].

##### Gluten

Some authors describe an association between the characteristic symptoms of FM and the presence of altered intestinal permeability and dysbiosis [6, 7, 10]. In the presence of dysbiosis occurs the destruction of tight juctions, proteins present in enterocytes and responsible for preventing the entry of pathogens. The consequent intestinal hyperpermeability triggers, in turn, an immunological reaction of inflammatory character [35], described by several authors as low grade inflammation [36]. Intestinal hyperpermeability appears to be caused by several factors, including gliadin present in gluten [37–39]. In this way, it would be possible to suggest that the exclusion of gluten could allow a lower prevalence of dysbiosis, and therefore less intestinal inflammation.

##### Dairy

There are several different casein subtypes in milk. In bovine milk, the predominant subtype is α-casein (50–55%), which does not exist in human milk [40], besides β-casein (35%) and κ-casein (15%). In addition, there are two types of β-casein, namely A1 and A2, being the A1 β-casein the prevalent one in Europe dairy products [41]. A systematic review concluded that A1 was associated to a higher prevalence of GI symptoms and increased intestinal inflammation in humans, compared to A2 [42]. The mechanism seems to be related to the activation of the Th2 signaling pathway in the intestine [43], which promote inflammation.

##### Sugar

Sugar is a recognizably inflammatory food. In recent years, WHO has been setting up standards for reducing its ingestion. Its excessive consumption promotes the production of free radicals, leading to an increase in oxidative stress [44]. On the other hand, a hyperinsulinogenic environment enhances the expression of pro-inflammatory molecules [45].

##### Ultra-processed foods

Several authors define ultra-processed food as potentially inflammatory, mainly due to its free sugars, hydrogenated fat, and food additives content [46, 47]. Additionally, it is known that its relevant accumulation of advanced glication products (AGEs) is also related to a pro-inflammatory effect [48, 49]. When ingested, AGEs cross the epithelial barrier, attaching to the receptors in the dendritic cells of the mucosa, and promote the uptake of the antigens and to T cells, specifically Th1, Treg, Th2, and Th17, pro-inflammatory and inducers of allergic process [50]. AGEs in the cell activate cascades of signaling the production of inflammatory molecules, such as TNFα, IL6, and VCAM1 [51].

##### Anti-inflammatory food components

There are some foods with recognized anti-inflammatory potential. Omega 3, especially at an adequate omega 6:omega 3 ratio, allows the production of prostaglandins, leukotrienes, resolvins, and protectins, which in turn promote the expression of anti-inflammatory cytokines [23]. In that sense, the ingestion of walnuts and omega 3 rich fish, such as tuna, mackerel, sardines, horse mackerel, and salmon, are being encouraged. Additionally, antioxidants in foods are known to decrease the free radicals production, which in turn helps to decrease the oxidative stress and, consequently, the expression of pro-inflammatory molecules [24, 52]. Thus, the intake of foods rich in antioxidants, such as fruit and vegetables, is also being promoted. Thus, the variability in the choice of vegetables and fruits was promoted, in order to obtain several different antioxidants, such as vitamin C (kiwi, orange), phenolic compounds (black grapes, pomegranate, blackberries, and raspberries), quercitin (apple), zeaxanthin (blueberries), indole-3-carbinol (broccoli, cabbage), and vitamin A (pumpkin, carrot, sweet potato). The intake of other foods rich in antioxidants was also promoted, such as cocoa, ginger, and white and green tea [53, 54].

Moreover, one of the most important factors in an anti-inflammatory diet is the maintenance of glycemic index, through a greater intake of fibers and suitable proteins and fats, against a balanced intake of carbohydrates.

#### Low FODMAPs diet

The presence of dysbiosis [4, 10, 12], and in particular of SIBO [8, 9], has been described in FM patients, with a significant improvement in pain, fatigue, gastric pain, mobility, and GI symptoms, after 4 weeks of low FODMAPs diet [11]. Marsh and colleagues meta-analysis support the efficacy of a diet with a low intake of foods rich in FODMAPs for a period of 4 to 6 weeks in the treatment of GI symptoms, including abdominal pain, abdominal distention, constipation, diarrhea, and flatulence [8], symptoms that are found very often in FM patients [4]. Since this is a recurrent situation in FM [4], it makes sense to start by trying to optimize the quality of the intestinal microbiota, in order to normalize these symptoms, before starting the anti-inflammatory diet.

This intervention involves avoiding all dairy; all cereals except rice and oats; cashew; all fruit other than banana, citrus, pineapple, red berries, strawberries, and kiwi; and all vegetables other than pumpkin, cabbage, lettuce, tomato, carrot, and cucumber, for a period of 4 to 6 weeks [8].

#### Control group

The control group is receiving a dietary meal plan based on healthy eating recommendations in accordance with WHO guidelines. According to WHO, a healthy diet contains at least 400 g of fruits and vegetables, excluding potatoes, sweet potatoes, cassava, and starchy roots. A consumption of legumes, nuts, and whole grains (wheat, maize, millet, oats, rice, rye) is also promoted, as well as an intake of less than 5 g of salt per day, less than 10% of total energy intake from free sugars and less than 30% of total energy intake from fats, giving preference to unsaturated fats [55].

#### Outcome measures

The primary PRO of interest for this study are pain, fatigue, quality of sleep, quality of life, GI symptoms, and the presence of inflammation. To determine the effect of dietary intervention on the disease, the following questionnaires are being included:

- Revised Fibromyalgia Impact Questionnaire (FIQR) [56], to verify the impact of FM on the patient’s life;
- Visual analog pain scale (EVA_Pain) [57], validated by Boonstra and colleagues [58], and brief pain inventory (BPI) to assess pain [59], validated by Keller and colleagues [60];
- Visual analog scale from a list of common gastrointestinal and extraintestinal symptoms in FM, IBS, and non-celiac gluten sensitivity (NCGS) to assess GI symptoms [61], validated by Bengtsson and colleagues [62];
- Short Form 36 (SF-36) [63], to check the quality of life, validated by Fredheim and colleagues [64];
- Validated Fatigue Severity Survey (FSS) [65], to check the fatigue level;
- Validated Pittsburg Sleep Quality Index (PSQI) [66], to check the quality of sleep.

Additionally, serum high-sensitive CRP (hs-CRP), Erythrocyte Sedimentation Rate (ESR) and Interleukin-8 (IL-8) are being measured to assess the presence of inflammation. The serum collection and hs-CRP and ESR analysis is being perfomed by *Joaquim Chaves Saúde* Laboratory, an external entity. The biomarker IL-8 quantification is being performed according to ImmuliteR© (Siemens, Germany) manufacturer’s protocol. Details on collection, laboratory evaluation, and storage of IL-8 is presented on Additional file 4.

Data on age, physical activity, and anthropometric parameters, such as waist circumference, height, and weight, are also being collected. Body composition, specifically fat mass, lean mass, and water, is being assessed by bio-impedance, through the scale of Inbody brand, model 770.

#### Patient and public involvement

Patients or members of the public were not involved in the design, conduct, reporting, or dissemination of the research.

### Statistical analysis

Baseline demographic and clinical characteristics of the participants of both groups will be analyzed using descriptive statistics. For the continuous normal distributed variables, the t-student test will be used to assess the association between the disease evaluation parameters, the inflammatory markers, and the dietary intervention. Correlations between variables will be sought at the different assessment moments. Regression coefficients will be calculated to determine the contribution of the domains for each variable. ANOVA will be used to evaluate the participants’ evolution within each group.

Missing data will not be included in the statistical analysis. Participants who discontinue or deviate from intervention protocols, as well as patients who meet exclusion criteria at some point of the intervention period, will be excluded. Motive of exclusion will be the outcome to be collected from these participants.

Statistical analysis will be performed using IBM SPSS Statistics Software, version 19.0. A *p* value of 0.05 is considered statistically significant.

## Discussion

The results of this study are expected to determine whether a change in patient nutrition helps to alleviate symptoms, which would optimize medical intervention.

To our knowledge, a nutritional approach involving a combination of several anti-inflammatory dietary factors has never been designed. Nutritional approaches in FM, to date, had always isolated dietary components that are believed to have a negative effect on disease symptoms, such as the application of a gluten-free [67] and aspartame-free diet [68]. An integrative approach has never been undertaken to include anti-inflammatory components and exclude the pro-inflammatory ones.

A recent systematic review (2018) allowed us to determine that dietary interventions seem to be promising as complementary therapies in FM, particularly a hypocaloric diet [27, 28], a raw vegetarian diet [25, 26], or a low FODMAP diet [11]. However, the studies that exist are of poor quality, according to the Cochrane Risk of Bias [29]. In our study, we intend to increase the sample size and ensure a good completion of nutritional interventions, in order to increase the quality of the study.

The WHO recommendations in the control group, which already could have some positive impact on patients’ health, could be a limiting factor in the interpretation of the results. However, once FM is associated with low-grade inflammation, those dietary recommendations per se may not be anti-inflammatory enough.

## Trials status

This is the first trial Protocol version, submitted on July 13, 2020. The trial is currently ongoing. Recruitment started on April 9, 2019, and will end in February 2021. We expect the end of the study to take place by April 2021.

## Supplementary Information

**Additional file 1.** SPIRIT 2013 Checklist: Recommended items to address in a clinical trial protocol and related documents*

**Additional file 2.** Informed Consent Statement.

**Additional file 3.** FODMAPs diet support table for patients.

**Additional file 4.** Interleukin-8 collection procedures.

## Data Availability

Anonymized dataset will be stored in Google Drive, available to all authors. Any data required to support the protocol can be supplied on request.

## Abbreviations

AGE: Advanced glycated product
BPI: Brief pain inventory
GI: Gastrointestinal
ESR: Eritrocyte sedimentation rate
EVA-Pain: Visual analog pain scale
EVA-GI: Visual analog scale from a list of common gastrointestinal and extraintestinal symptoms in FM
FIQ-R: Revised Fibromyalgia Impact Questionnaire
FM: Fibromyalgia
FODMAPs: Fermentable oligo-, di-, and monosaccharides and polyols
FSS: Fatigue severity survey
hs-CRP: Serum C-reactive protein
IBS: Irritable bowel syndrome
IL: Interleukin
IPR: Instituto Português de Reumatologia
NSAID: Non-steroid anti-inflammatory drugs
PRO: Patient’s reported outcomes
PSQI: Pittsburg sleep quality index
SF-36: Short Form 36
SIBO: Small intestinal bacterial overgrowth
WHO: World Health Organization
