# Supplementary material for: A study protocol for a randomized controlled trial of an anti-inflammatory nutritional intervention in patients with fibromyalgia
Source: Trials. 2021 Mar 9;22:198. doi: 10.1186/s13063-021-05146-3 (PMC7944600; doi:10.1186/s13063-021-05146-3)
Supplement: Supplementary file 2 — Additional file 2. Informed Consent Statement. [file 13063_2021_5146_MOESM2_ESM.pdf]

Informed Consent Statement

I express my agreement and spontaneous willingness to participate in the study with the theme "Effects of a nutritional intervention poor in potentially inflammatory components in the parameters of assessment of the disease, inflammatory markers and quality of life of patients with Fibromyalgia".

I realized that this study aims to analyse the effects of a potentially anti-inflammatory diet that is low in foods with a high content of FODMAPs (foods more fermentable by the small intestine), and a World Health Organization (WHO) Healthy diet, in reducing the most common symptoms of Fibromyalgia, namely pain, fatigue, quality of sleep and gastrointestinal changes.

The anti-inflammatory diet is characterized by being free from foods considered pro-inflammatory, such as gluten, dairy products, sugar and ultra-processed products, with a preference for eating vegetables, fruits, meat, fish and eggs, rice, potatoes, oats, nuts and oilseeds. During the first month of intervention, the group of patients who integrate the anti-inflammatory diet, will also have to eat a diet low in foods with a high content of FODMAPs, thus excluding all dairy products; all cereals except rice; cashew; all fruits with the exception of bananas, citrus fruits, pineapples, red fruits, strawberries and kiwi; all vegetables except pumpkin, cabbage, lettuce, tomato, carrot and cucumber. After this period, all excluded vegetables and fruits will be reintroduced, maintaining an anti-inflammatory diet for 2 months, making a total of 3 months of intervention. The WHO Healthy diet is characterized by a high intake of vegetables and fruits, moderate intake of legumes, whole grains, fish, meat and dairy products, and reduced consumption of saturated fat, salt and sugar.

As a participant, I will be prescribed a personalized dietary plan, according to my food preferences and energy needs, and that meets the proposed requirements, which I must follow for a consecutive period of 3 months. I will be followed up by telephone contact every fifteen days, and I will be able to take any questions that arise through the contact provided. At the beginning and at the end of the nutritional intervention, I will have to answer a set of questionnaires related to the disease, and blood will be collected for analysis of inflammatory biomarkers.

I am aware that my participation in this study is voluntary and that the non-participation will not do me any harm. If I wish to interrupt the study, my consent will not be impaired. There will be no monetary bonus for participating in the study. The information collected is intended

for statistical treatment and publication only, and will be handled by the advisors and their representatives. Collection is anonymous and confidential.

It is hoped that the results of this study will determine whether a change in patients diet helps to alleviate symptoms, which would optimize medical intervention.

I **ACCEPT** participating in this study, confirming that I have been informed about the conditions of the study and that I have no doubts. I **AGREE** to follow the recommended diet, answer all questionnaires and perform clinical analyses before and after the intervention.

Date: ___/___/______

Name: ____

Signature: ____

(Signature of the participant or, in the case of minors, the parent or legal guardian)

I discussed treatment with the patient mentioned above.

Date: ___/___/______

Signature of the responsible: ____
