# Supplementary material for: A study protocol for a randomized controlled trial of an anti-inflammatory nutritional intervention in patients with fibromyalgia
Source: Trials. 2021 Mar 9;22:198. doi: 10.1186/s13063-021-05146-3 (PMC7944600; doi:10.1186/s13063-021-05146-3)
Supplement: Supplementary file 3 — Additional file 3. FODMAPs diet support table for patients. [file 13063_2021_5146_MOESM3_ESM.pdf]

VEGETAIS A EVITAR

Alho
Alho-francês
Cebola
Chalotas
Espargos
Beterraba
Leguminosas (feijão, grão, ervilhas, lentilhas, favas)
Brócolos
Couve-flor
Repolho
Beringela
Agrião
Cogumelos
Soja e rebentos de soja
Batata doce
Hummus (mesmo caseiro)
Molho pesto
Picles

VEGETAIS A PREFERIR

Alface
Rúcula
Couve coração
Cenoura
Abóbora
Rabanete
Pepino
Tomate
Chuchu
Curgete (sem casca)
Espinafres
Aipo
Batata
Nabo
Pimento

FRUTAS A EVITAR

Maçã
Pêra
Pêssego
Nectarina
Figo
Uva e passas de uva
Cereja
Ameixa
Melancia, meloa, melão
Manga
Romã
Abacate
Bagas goji

FRUTAS A PREFERIR

Banana
Kiwi
Citrinos: laranja, tangerina, limão
Abacaxi e ananás
Frutos vermelhos: framboesa, mirtilos e amoras, arandos (cranberries)
Morangos
Papaia
Coco

CEREAIS A EVITAR

Trigo (pão, bolos, biscoitos, bolachas, massa, massa folhada, salgados, pastelaria, panados, cereais de pequeno-almoço, barras de cereais, couscous),
Kamut e Espelta
Centeio
Cevada
Amaranto
Muesli e granola de compra (com açucar, mel)

CEREAIS A PREFERIR

Arroz
Aveia
Quinoa

OLEAGINOSAS A EVITAR

Caju
Amêndoa
Farinha de amêndoa
Pistacho

ADOÇANTES E TEMPEROS

Agave
Fructose
Syrup de milho ou maçã
Mel
Produtos com zero açucares, que contenham polióis (geralmente aditivos que terminam em –ol ou isomaltose)
Adoçantes (inulina, isolmaltose, malitol, manitol, sorbitol, xilitol)

OLEAGINOSAS A PREFERIR

Noz
Avelã
Pinhões
Sementes (girassol, sésamo, chia, linhaça, abóbora)
Manteiga de amêndoa, caju, avelã, sésamo

ADOÇANTES E TEMPEROS

Vinagre de cidra
Cacau em pó magro
Canela
Açúcar de coco
Azeite
Óleo de coco
