# Supplementary material for: A study protocol for a randomized controlled trial of an anti-inflammatory nutritional intervention in patients with fibromyalgia
Source: Trials. 2021 Mar 9;22:198. doi: 10.1186/s13063-021-05146-3 (PMC7944600; doi:10.1186/s13063-021-05146-3)
Supplement: Supplementary file 4 — Additional file 4. Interleukin-8 collection procedures. [file 13063_2021_5146_MOESM4_ESM.pdf]

# INTERLEUKIN-8 COLLECTION PROCEDURES

## OBJECTIVE

This statement aims to provide details about collection, storage and laboratory evaluation of Interleukin-8 (IL-8).

## INTRODUCTION

Immunoassay was the method chosen to detect and measure the amount of IL-8 in serum of Fibromyalgia patients belonging to randomized controlled trial of an anti-inflammatory nutritional intervention. Immulite© (Siemens, Germany) technology will be used for the assay. Randox Elisa kit, specifically High-Sensitive Enzyme-linked Immunosorbent Assay Kit for Interleukin-8 from BioNova Científica S.I. will be used.

## BLOOD COLLECTION

Serum collection is performed by Joaquim Chaves Saúde Laboratory. No special conditions are required for patient.
Informed consent were applied previously to the sample collection. Serum collection is anonymous. Each patient is given a code to ensure confidentiality. Identifiability is only possible by Sponsor, who is conducting the intervention.

## SAMPLE STORAGE

Samples are stored in a chest at -80ºC until the moment of analysis. Samples are being stored by Joaquim Chaves Saúde Laboratory. They will be delivered to the Biochemistry Laboratory of the Cooperativa de Ensino Superior Egas Moniz, where the analysis will take place.
According to High-Sensitive Enzyme-linked Immunosorbent Assay Kit for Interleukin-8 (IL-8) Instruction Manual [1], freeze/thaw cycles should be avoid. Hemolytic specimen should not be used, since it could influence the results.

## STORAGE OF THE KITS

Reagents and materials provided by High-Sensitive Enzyme-linked Immunosorbent Assay Kit for Interleukin-8 are:

1. Pre-coated, ready to use 96-well strip plate (1 unit)
2. Plate sealer for 96 wells (4 units)
3. Standard (2 units)
4. Standard Diluent (1×20mL)
5. Detection Reagent A (1×120μL)
6. Assay Diluent A (1×12mL)
7. Detection Reagent B (1×120μL)
8. Assay Diluent B (1×12mL)
9. TMB Substrate (1×9mL)
10. Stop Solution (1×6mL)
11. Wash Buffer (30 × concentrate) (1×20mL)

According to High-Sensitive Enzyme-linked Immunosorbent Assay Kit for Interleukin-8 (IL-8) Instruction Manual [1], the Standard, Detection Reagent A, Detection Reagent B and the 96-well strip plate will be stored at -20ºC upon receipt while the others will be at 4ºC.

## SAMPLE ANALYSIS METHODOLOGY

Immulite© (Siemens, Germany) will be used to analyze IL-8. The immunoassay will occur in the Biochemistry Laboratory of the Cooperativa de Ensino Superior Egas Moniz. The analysis will be performed by some of the authors, namely ARS, MLS, AB and MFM.

Sample analysis methodology is described in detail, according to High-Sensitive Enzyme-linked Immunosorbent Assay Kit for Interleukin-8 (IL-8) Instruction Manual [1].

### Reagents preparation

1. All kit components and samples should be at 18-25ºC before use.

2. Standards will be prepared within 15 minutes before assay. Reconstitution of the Standard will be done with 1.0mL of Standard Diluent, kept for 10 minutes at 18-25ºC, gently shaking (not to foam). The concentration of the Standard in the stock solution is 500pg/mL. Firstly, dilution of the stock solution to 250pg/mL will be made, which serves as the highest Standard (250pg/mL). Then, seven tubes containing 0.5mL of Standard Diluent will be prepared, and the diluted Standard will be used to produce a double dilution series: 250pg/mL, 125pg/mL, 62.5pg/mL, 31.2pg/mL, 15.6pg/mL, 7.8pg/mL, 3.9pg/mL, and 0pg/mL (the blank).
3. Detection Reagent A and Detection Reagent B will be briefly centrifuged before use. Dilution to the working concentration 100-fold with Assay Diluent A and B, respectively, will be made.
4. 20mL of Wash Solution concentrate (30×) will be diluted with 580mL of deionized or distilled water to prepare 600mL of Wash Solution (1×).
5. The needed TMB substrate dosage of the solution will be aspirated with sterilized tips.

If crystals have formed in the Wash Solution concentrate (30×), reagent should be warm to 18-25ºC and gently mixed until the crystals are completely dissolved.

**Sample preparation**

Optimal sample dilutions determination will be made, according to a suggested 50-fold dilution: 10μL Sample + 490μL PBS. Sample will be diluted by 0.01mol/L PBS (PH=7.0-7.2).

**Assay procedure**

1. According to reagents preparation, 7 wells for diluted standard, blank and sample will be prepared. 100μL of each dilutions of standard, blank and samples will be added into the appropriate wells. Wells will be covered with the Plate sealer and incubated for 1 hour at 37º C.
2. Liquid of each well will be removed without washing.
3. 100μL of Detection Reagent A working solution will be added to each well. Wells will be covered with the Plate sealer and incubated for 1 hour at 37º C.
4. The solution will be aspirated and washed with 350μL of 1× Wash Solution using a squirt bottle, multi-channel pipette, manifold dispenser or auto-washer to

each well, and rest for 1-2 minutes. The remaining liquid will be completely removed from all wells by snapping the plate onto absorbent paper. Total washing will be performed 3 times. After the last wash, the remaining Wash Buffer will be removed by aspirating or decanting, and the plate blotted against absorbent paper.

5. 100μL of Detection Reagent B working solution will be added to each well. Wells will be covered with the Plate sealer and incubated for 30 minutes at 37º C.
6. Aspiration/wash process will be repeated for total 5 times as conducted in step 4.
7. 90μL of Substrate Solution will be added to each well. Wells will be covered with a new Plate sealer and incubated for 10 to 20 minutes at 37º C, protecting from light. The liquid will turn yellow by the addition of Substrate Solution.
8. 50μL of Stop Solution will be added to each well. The liquid will turn yellow by the addition of Stop solution. The liquid will be mixed by tapping the side of the plate, until the colour appears uniform.
9. Any drop of water and fingerprint will be removed from on the bottom of the plate. Any bubble on the surface of the liquid will be removed. Then, microplate reader will be initiated and measurement at 450nm will be conducted immediately.

According to High Sensitive Enzyme-linked Immunosorbent Assay Kit for Interleukin-8 (IL-8) Instruction Manual [1], the microplate provided has been pre-coated with an antibody specific to IL-8. Standards or samples are then added to the appropriate microplate wells with a biotin-conjugated antibody specific to IL-8. Next, Avidin conjugated to Horseradish Peroxidase (HRP) is added to each microplate well and incubated. After TMB substrate solution is added, only those wells that contain IL-8, biotin-conjugated antibody and enzyme-conjugated Avidin will exhibit a change in colour. The enzyme-substrate reaction is terminated by the addition of sulphuric acid solution and the colour change is measured spectrophotometrically at a wavelength of 450nm ± 10nm. The concentration of IL-8 in the samples is then determined by comparing the optical density (O.D.) of the samples to the standard curve.

# REFERENCES


1. Corporation, C.-C. *High Sensitive Enzyme-linked Immunosorbent Assay Kit for Interleukin-8 (IL-8) Instruction Manual*. 5th edition:[Available from: http://www.cloud-clone.com/manual/High-Sensitive-ELISA-Kit-for-Interleukin-8-(IL8)-HEA080Hu.pdf.
